# Linear incrementality in focus and accentuation processing during sentence production: evidence from eye movements

**DOI:** 10.3389/fnhum.2024.1523629

**Published:** 2025-01-07

**Authors:** Zhenghua Zhang, Qingfang Zhang

**Affiliations:** Department of Psychology, Renmin University of China, Beijing, China

**Keywords:** sentence production, focus and accentuation processing, linear incrementality, hierarchical incrementality, eye movements

## Abstract

**Introduction:**

While considerable research in language production has focused on incremental processing during conceptual and grammatical encoding, prosodic encoding remains less investigated. This study examines whether focus and accentuation processing in speech production follows linear or hierarchical incrementality.

**Methods:**

We employed visual world eye-tracking to investigate how focus and accentuation are processed during sentence production. Participants were asked to complete a scenario description task where they were prompted to use a predetermined sentence structure to accurately convey the scenario, thereby spontaneously accentuate the corresponding entity. We manipulated the positions of focus with accentuation (initial vs. medial) by changing the scenarios. The initial and medial positions correspond to the first and second nouns in sentences like “N1 is above N2, not N3.”

**Results:**

Our findings revealed that speech latencies were significantly shorter in the sentences with initial focus accentuation than those with medial focus accentuation. Furthermore, eye-tracking data demonstrated that speakers quickly displayed a preference for fixating on initial information after scenarios onset. Crucially, the time-course analysis revealed that the onset of the initial focus accentuation effect (around 460 ms) preceded that of the medial focus accentuation effect (around 920 ms).

**Discussion:**

These results support that focus and accentuation processing during speech production prior to articulation follows linear incrementality rather than hierarchical incrementality.

## 1 Introduction

To achieve successful communication, speakers must transform their communicative intentions into coherent utterances through a specific sequence of words. This process involves conceptualization, linguistic encoding (including grammatical and prosodic encoding), and articulation (Ferreira, 1993, 2010; Konopka and Kuchinsky, 2015; Levelt, 1989). Most models of language production assume that message planning and linguistic encoding proceed incrementally (Levelt, 1989). That is, speakers do not need to wait until planning is complete before moving on to the next stage, but only need to plan a fragment of the complete utterance before progressing to subsequent stages. This incremental approach allows speakers to initiate articulation before full planning, with the remaining aspects of the utterance being constructed “on-the-fly” after speech onset (e.g., Brown-Schmidt and Konopka, 2008; Konopka and Kuchinsky, 2015; Levelt, 1989; Smith and Wheeldon, 1999). While considerable research in language production has focused on incremental processing during conceptual and grammatical encoding (e.g., Garrett, 1980; Konopka and Meyer, 2014; Konopka and Kuchinsky, 2015), prosodic encoding remains less investigated. This study aims to explore the incremental processing in conceptualization and prosodic encoding during the production of sentences with focus accentuation. Before presenting our experiment, we review relevant theoretical and empirical studies concerning the incremental processing in conceptual and linguistic encoding as well as the relation between focus and accentuation during speech production.

Conceptualization, or message formulation involves converting a communicative intention into a preverbal semantic representation. This involves gathering information at the message-level, including details about the entities involved in an event, the relationships between these entities (i.e., the gist of event, for example, who-did-what-to-whom), and the overall type of message being conveyed. Linguistic encoding in language production involves both grammatical and prosodic encoding. Grammatical encoding concerns accessing lemmas and constructing of syntactic structure, while prosodic encoding entails generating prosodic constituents such as prosodic words, phonological phrases, and intonational phrases. This is followed by establishing a metrical grid based on the prosodic constituent structure (Ferreira, 1993; Levelt, 1989).

There are two predominant theoretical frameworks concerning the incrementality process about conceptualization and linguistic encoding in sentence production. The first framework, termed linear incrementality (e.g., Brown-Schmidt and Konopka, 2008; Ganushchak et al., 2017; Gleitman et al., 2007; Meyer and Meulen, 2000), assumes that speakers can prepare a sequence of small conceptual and linguistic increments without relying on a complete conceptual framework. Accordingly, the initial increment of conceptual and linguistic encoding may consist of only information pertinent to a single entity, typically the one mentioned first by speakers. In contrast, the second framework, termed hierarchical incrementality (e.g., Bock et al., 2004; Griffin and Bock, 2000; Hwang and Kaiser, 2014; Kuchinsky and Bock, 2010; Lee et al., 2013; Momma et al., 2016), assumes that speakers do not encode concepts and words individually; rather, they initially formulate the gist of an event and establish a conceptual framework during conceptualization. Subsequently, during linguistic encoding, they first construct a syntactic framework to organize the target sentence.

The majority of studies using eye movement technology have consistently shown that speakers preferentially encode the entity mentioned first in the speech, thus providing support for the linear incrementality hypothesis (Ganushchak et al., 2017; Gleitman et al., 2007; Griffin, 2001; Meyer and Meulen, 2000; Konopka and Meyer, 2014; Schlenter et al., 2022). Gleitman et al. (2007) used the attention capture paradigm to manipulate visual salience and found that the most visually salient character was consistently assigned to the initial position of the sentence. Crucially, speakers fixated on this character preferentially within 200 ms of scenario onset and maintained their fixation until speech onset. Similar findings were reported by Ganushchak et al. (2017), who instructed participants to construct active sentences (e.g., “The frog catches the fly.”) during a scenario description task. They manipulated the accessibility of agent and patient words by providing varying levels of semantic context information and related vocabulary. It was observed that speakers prioritized fixating on the agent very rapidly after the presentation of the picture (within 400 ms). Moreover, the availability of agent words was found to influence fixation patterns on the scenario after 400 ms, suggesting that speakers predominantly encode this character rather than formulating a comprehensive conceptual framework encompassing information about both agents and patients.

In contrast, several studies provide empirical support for hierarchical incrementality (e.g., Bock et al., 2004; Griffin and Bock, 2000; Hwang and Kaiser, 2014; Kuchinsky and Bock, 2010; Lee et al., 2013; Momma et al., 2016). Griffin and Bock (2000) conducted a study in which participants were presented with scenarios, and their eye movements were tracked while performing either a linguistic task (describing the scenario) or a non-linguistic task (detecting a patient). The findings revealed intriguing observations: within the initial 300 ms of scenario presentation, participants did not exhibit a fixation preference toward any specific entity, irrespective of the task. However, beyond this initial period, participants exhibited dictinct fixation patterns depending on the task at hand. In the scenario description task, participants predominantly fixated on the subject entity after the initial 300 ms, whereas in the patient detection task, their fixation was directed toward the patient entity. These findings suggest that speakers rapidly grasp the essence of an event, such as who performed an action on whom, and generate a conceptual framework within ~300 ms after scenario onset. Subsequently, they shift their gaze to the entity that is established as the appropriate starting point based on the conceptual framework, thus leading support to hierarchical incrementality during conceptualization.

Additionally, Do and Kaiser (2019) discovered evidence supporting hierarchical incrementality in linguistic encoding. In contrast to the approach taken by Ganushchak et al. (2017), who employed SVO (Subject-Verb-Object) sentences where the hierarchical syntactic structure and the surface linear word order of the utterance are isomorphic, Do and Kaiser (2019) utilized object questions in English to separate the syntactic subject from the linearly initial word. Participants were instructed to produce declarative sentences (e.g., “The nurses tickled the maids.”) and object questions in English (e.g., “Which maids did the nurses tickle?”), with a syntactic subject (i.e., the nurses) placed in the initial or medial position within the sentence. The findings revealed an initial preference for fixations to the subject region ~400 ms after scenario onset, despite the subject not being the linearly initial element in object questions. This suggests that speakers construct a syntactic frame and then assign a concept to serve as the structurally-initial element of the utterance. Notably, speakers shifted their fixation to the object relatively soon after fixating on the subject in object questions, indicating that linear and hierarchical incrementality are distinct yet closely coordinated facets of sentence encoding.

Indeed, studies examining the online processes of conceptual and linguistic encoding confirm that the incremental processing strategy at both levels may exhibit considerable variability, particularly in response to the ease of conceptual and linguistic encoding (Konopka and Meyer, 2014; Konopka and Kuchinsky, 2015; Konopka, 2012; Kuchinsky and Bock, 2010). Konopka and Meyer (2014), for instance, manipulated the ease of conceptual encoding through character codability (variations in speakers' noun selection) and event codability (variations in verb choice), while also manipulating the ease of linguistic encoding through lexical primes (words semantically or associatively related to a character) and structural primes (active and passive syntax) to investigate sentence production. Their findings indicated that speakers are inclined to encode individual message elements sequentially when one character is easy to identify or when the event is complex to comprehend. Conversely, they are more likely to prioritize encoding information about both characters when employing a more familiar syntactic structure or when the event is straightforward to encode. Similar findings were replicated in the study conducted by Konopka and Kuchinsky (2015), where they observed that speakers exhibited a more consistent allocation of attention to both characters during linguistic encoding in structurally primed sentences compared to unprimed ones. However, they also noted that the priming effects on eye movements were attenuated by conceptual familiarity. This suggests speakers are inclined to prepare larger message increments when the sentence structure is primed, albeit this tendency is constrained by message-level information. The variability in increment size observed during conceptual and linguistic encoding aligns with the claim that incrementality represents an adaptive feature of the production system, allowing speakers to employ diverse planning strategies (Jaeger, 2010).

It is noteworthy that while previous studies have predominantly focused on grammatical encoding in sentence production, empirical investigations into prosodic encoding have been relatively limited. Currently, research has begun to investigate the planning scope of phonological encoding, revealing that it may extend to the first prosodic word (Meyer, 1996; Schriefers, 1999; Jescheniak et al., 2003; Wheeldon and Lahiri, 1997). Phonological encoding includes not only prosodic encoding but also the retrieval of segmental information (Levelt, 1989). Meyer (1996) conducted a study wherein participants were presented with pairs of pictures described by noun phrases (e.g., “the arrow and the bag”) or sentences (e.g., “the arrow is next to the bag”), accompanied by an auditory distractor word phonologically related to either the first or second noun, or unrelated to both. The findings revealed that when the distractor word was phonologically related to the first noun, speech latency was shortened, whereas no such phonological facilitation was observed for the second noun. This suggests that prior to articulation, the form of the first noun is already selected, and the planning scope of phonological encoding may extend to the first prosodic word. A similar conclusion was drawn in the study of Wheeldon and Lahiri (1997). They initially presented participants with a visually displayed noun phrase or adjective phrase, followed by an auditorily presented a question related to that phrase. Participants were then instructed to formulate a sentence in response to the question using the words they had seen as soon as possible. The results revealed that speech latency was contingent upon the complexity of the first prosodic word.

The conventional perspective once held that syntactic structure directly dictates the prosodic features of a sentence (Cooper and Danly, 1981; Klatt, 1975; Selkirk, 1984). However, more recent evidence suggests the presence of prosodic representations that are relatively autonomous from syntactic structure and directly shape prosodic features (e.g., Ferreira, 1993; Jun and Bishop, 2015; Jungers et al., 2016; Tooley et al., 2018; Wheeldon and Lahiri, 1997; Zhang and Zhang, 2019). These studies have revealed that prosodic structures do not align perfectly with syntactic structure (Ferreira, 1993; Gee and Grosjean, 1983; Miyamoto and Johnson, 2002). Additionally, researchers also observed prosodic features such as speech rate, prosodic boundaries, and rhythm, which exhibit priming effects, suggesting the presence of independent representations for these prosodic features (Jun and Bishop, 2015; Jungers et al., 2002; Jungers and Hupp, 2009; Jungers et al., 2016; Tooley et al., 2014, 2018; Zhang and Zhang, 2019). Therefore, it is necessary to investigate the incremental processing strategy in prosodic encoding.

We hypothesize that the incremental processing strategy of prosodic encoding could follow either linear incrementality or hierarchical incrementality. Under the framework of linear incrementality, speakers generate smaller units (e.g., prosodic words) sequentially in the order of mention. In contrast, under the framework of hierarchical incrementality, speakers construct a prosodic frame at the sentence level first, which then guides subsequent production.

The reasons for proposing these two hypotheses are as follows: First, prosody is closely linked to syntax (Cooper and Danly, 1981; Klatt, 1975; Selkirk, 1984) and may directly connect to message-level representations (Levelt, 1989; Tooley et al., 2018). Previous studies have demonstrated that conceptualization and linguistic encoding in sentence production follow either linear or hierarchical incrementality. Thus, it is plausible that prosodic encoding might share similar patterns.

Second, evidence from prior studies supports these two potential strategies. On the one hand, word-level prosodic encoding research supports linear incrementality. That is, speakers access stress on the first syllable before the second (Schiller et al., 2004, 2006; Schiller, 2006). Stress refers to the relative prosodic prominence of one syllable compared to others (Cutler, 1984). Schiller et al. (2004) found that words with initial stress are produced faster than those with final stress. This pattern was further corroborated by lexical stress monitoring tasks and go/no-go decision tasks (Schiller et al., 2006; Schiller, 2006). Additionally, studies have indicated that the planning scope of phonological encoding may extend to the first prosodic word (Meyer, 1996; Jescheniak et al., 2003). These findings suggest that prosodic encoding may align with linear incrementality.

On the other hand, some research findings suggest that prosodic encoding might follow a hierarchical incremental pattern. Speech error studies have shown that the overall accentuation pattern remains stable even when words are swapped during phrase production (Garrett, 1975, 1976). Similarly, acoustic analyses of sentence recall tasks found that while the duration of individual words varied, the overall rhythmic structure of the sentence remained consistent (Ferreira, 1993). Additionally, priming studies have revealed prosodic priming effects at both the word and sentence levels, such as stress, speech rate, prosodic boundaries, and rhythm (e.g., Jungers et al., 2016; Tooley et al., 2014, 2018; Yu et al., 2024; Zhang and Zhang, 2019), supporting the existence of prosodic frames at multiple levels.

In daily communication, speakers often use accentuation, a prosodic feature, to highlight important information (referred to as focus) within an utterance, thereby enhancing the accuracy and effectiveness of information delivery. Focus typically pertains to new or contrasting information at the message-level (Chomsky, 1971; Gundel et al., 1993). Although our current understanding of the role of focus during real-time language production is somewhat limited, it is generally believed that focus is specified in conceptualization (Ferreira, 1993; Ganushchak et al., 2014; Levelt, 1989; Tooley et al., 2018). Previous studies have primarily employed question–answer pairs to establish a focus context, where the interrogative words and key answers corresponding to the questions are focus information (e.g., Chen et al., 2015; Dimitrova et al., 2012; Do and Kaiser, 2019; Ganushchak et al., 2014; Magne et al., 2005).

Studies have shown that focus may influence sentence production. Ganushchak et al. (2014) used the visual world eye-tracking paradigm to investigate the fixation patterns during the production of declarative sentences under contexts with varying focus positions in Dutch and Chinese. They used questions to create a focus context, and after hearing a question, participants were instructed to answer questions with complete declaratives according to scenarios. This is exemplified in the following examples:

Neutral-focused: “What is happening here?” (in Dutch, “Wat gebeurt hier?”; in Chinese, “发生了什么？”).

Subject-focused: “Who is stopping the truck?” (in Dutch, “Wat stopt de politieman?”; in Chinese, “谁在停止卡车？”).

Object-focused: “What is the policeman stopping?” (in Dutch, “Wie stopt de vrachtauto?”; in Chinese, “警察在停止什么？”).

Answer: “The policeman is stopping the truck.” (in Dutch, “De politieman laat een vrachtauto stoppen.”; in Chinese, “警察在停止卡车。”).

The findings revealed that in both languages, after 400 ms of scenarios onset, speakers exhibited increased fixation on the focus information, and this fixation lasted longer when producing subject-focused or object-focused sentence compared to neutral-focused ones. This suggests that the focus context influences subsequent sentence production, facilitating speakers in rapidly and concurrently encoding focus information in various positions. In contrast, Do and Kaiser (2019) did not find evidence for the role of focus in speech production. In their study, participants were first provided with a letter cue and then asked to produce a sentence in Chinese based on a given scenario. If the cue was “s,” participants would describe the scenario with a declarative sentence (e.g., “The chefs shot the nurses.”), while a “q” cue would prompt the production of an object question (e.g., “The chefs shot which nurses?”). There is no significant difference in fixation patterns during the production of declarative sentences and object questions. The divergent findings might stem from differences in processing mechanisms and processing costs between questions and declaratives (Aoshima et al., 2004; Sussman and Sedivy, 2003), as proposed by Do and Kaiser (2019). Unlike the production of questions in Do and Kaiser (2019), the production of declarative sentences in Ganushchak et al. (2014) may also involve comprehending (or recalling) the question being answered. Additionally, the processing cost for declarative sentence production in Ganushchak et al. (2014) is likely lower than for question production in Do and Kaiser (2019), as participants in the former had already been provided with most of the relevant information in the question.

Focus is intricately linked to accentuation, a term that broadly describes the presence of prosodic prominence on a specific element within a sentence (Li et al., 2018). As a crucial prosodic feature, accentuation primarily undergoes processing during prosodic encoding (Ferreira, 1993; Levelt, 1989). In Chinese, as a tonal language, accentuation is realized through pitch maximum raising, duration lengthening or intensity increasing (Li et al., 2008; Li and Yang, 2013; Liu and Xu, 2005; Xu, 1999). Numerous studies have found that focus is often marked with accentuation, while non-focus elements tend to be deaccented, as evidenced through phonetic analysis of spoken dialogues (Chen et al., 2015; Eady and Cooper, 1986; Patil et al., 2008; Pierrehumbert, 1993; Xu et al., 2012). Consequently, focus can be prosodically distinguished by accentuation across languages, including German (Burght et al., 2021), Mandarin (Chen et al., 2015), Dutch (Dimitrova et al., 2012), English (Dahan et al., 2002), French (Magne et al., 2005), and Japanese (Ito and Garnsey, 2004). Tooley et al. (2018) employed a priming paradigm to investigate whether accentuation operates an independent representation. In their experiment, participants were presented with a prime sentence featuring a specific accentuation pattern auditorily, followed by the visually presented target sentences. Subsequently, the target sentence disappeared, and participants were asked to recall it. Interestingly, the results indicated that the accentuation of prime sentences did not affect target sentences. Tooley et al. (2018) proposed that despite the absence of an accentuation priming effect, accentuation is widely assumed to be independently represented (Gussenhoven, 1983; Selkirk, 1995). They suggested that the absence of a priming effect could be attributed to accentuation encoding having direct communication with message-level representations, such as focus. Consequently, any priming effect might not be robust enough to survive the linguistic planning for subsequent target sentences. Notably, focus and accentuation are not always strictly on a one-to-one basis. The determination of focus position is constrained by contextual factors, but once the focus position is determined, the distribution of accentuation is constrained by the specific structural rules of the language (Gussenhoven, 1983; Ladd, 2008; Selkirk, 1995).

Although focus with accentuation is a prevalent part of naturalistic communication, it has received limited attention in the realm of real-time language production, leaving us uncertain about how to process focus and accentuation during speech production. Levelt (1989) proposed that speakers mark focus during the conceptualization, with this marking subsequently transforming into accentuation during prosodic encoding, potentially influencing the intonation of the eventual utterance. Furthermore, he posited that accentuation can proceed incrementally from left to right. However, the question of how focus information is processed remain unspecified, and these views lack empirical support until now. According to the principle of incrementality for sentence production, we hypothesize that the process of conceptualization and prosodic encoding generally adheres to this incremental principle.

According to the framework of linear incrementality, we assume that speakers can prepare a sequence of small conceptual and accentuation pattern increments. That is, speakers sequentially encode the conceptual and accentuation features of a single entity, following the order in which entities are expressed. Consequently, speakers are expected to prioritize processing the earlier mentioned focus and accentuation. According to the framework of hierarchical incrementality, we assume that speakers generate an overall conceptual framework along with a corresponding sentence-level accentuation pattern. Therefore, speakers should simultaneously process focus and accentuation regardless of its position in the mention sequence. These two frameworks primarily diverge in their views on the increment size of focus and accentuation processing. The former suggests that the increment size of focus and accentuation processing is small, such as the prosodic word, while the latter proposes that it extends to the overall sentence.

Given that focus can be specified during conceptualization and accentuation is processed during prosodic encoding (Ferreira, 1993; Ganushchak et al., 2014; Levelt, 1989; Tooley et al., 2018), we manipulated the position of focus and accentuation to investigate the interplay between conceptualization and prosodic encoding in sentence production. To investigate the incremental framework (linear incrementality vs. hierarchical incrementality) employed in focus and accentuation processing, we compared speech latencies and fixation patterns in sentences with different positions of accentuation. Previous studies typically employed question–answer pairs to induce focus accentuation (Chen et al., 2015; Xu et al., 2012). However, and this method may introduce a preview effect, potentially confounding the results of speech latencies (Allum and Wheeldon, 2009). This confusion is evident in the study of Ganushchak et al. (2014), which reported that speech latency in the medial focus is shorter than that in the initial focus. To address this, we employed the visual world paradigm, allowing speakers to spontaneously produce sentences with varying positions of focus with accentuation without the preview of information (see method section for further details) in the current study.

In addition, we used eye movement technology to capture fixation information on different entities displayed simultaneously on the same screen. This allowed us to examine the time-course of speech planning and assess how focus with accentuation at different positions affects the production of target sentences. More importantly, we aimed to distinguish between conceptualization and prosodic encoding based on the time course. The early time window (within about 400 ms) after the scenario onset corresponds to conceptualization, whereas the later time window (about 400 ms to speech onset) encompasses linguistic encoding, including grammatical and prosodic encoding (Ganushchak et al., 2014; Griffin and Bock, 2000; Schlenter et al., 2022; van de Velde et al., 2014; Konopka and Meyer, 2014). Given the lack of clarity regarding the timing of various processes involved in sentence production research, we adopted a data-driven approach to select time windows for analysis, aiming to provide more precise timing data.

Sentences with identical structures but differing accentuation positions (e.g., “The turtle is above the frog, not the peacock”; “The turtle is above the frog, not the peacock.” The underlined is the focus with accentuation), provide an ideal experimental setup for examining the mechanism employed in focus and accentuation processing. According to the linear incrementality, speakers are expected to prioritize encoding focus and accentuation occurring earlier in the mentioned positions, whereas the hierarchical incrementality predicts that speakers should simultaneously encode focus and accentuation in different mentioned positions.

We hypothesized that if the production of focus accentuation sentences follows linear incrementality, the speech latencies for sentences with initial focus accentuation will be significantly shorter than those with medial focus accentuation. Furthermore, participants in both conditions are expected to initially fixate on the information at the beginning of the sentence, followed by fixation on the information in the middle of the sentence. More importantly, we expect that the effect of initial focus accentuation will occur earlier in time compared to the effect of medial focus accentuation effect. At both positions, the presence of focus accentuation effect is expected to result in a greater proportion of fixation on the accented pictures compared to the deaccented pictures.

If the production of focus accentuation sentences follows hierarchical incrementality, we expect that there will be no difference in speech latencies between sentences with initial and medial focus accentuation. Furthermore, participants in both conditions are expected to initially exhibit no clear fixation preference toward any entity in scenario after its presentation, followed by rapidly fixate on the accented pictures. Critically, there should be no differences in the timing of the focus accentuation effect between initial and medial positions.

## 2 Method

### 2.1 Participants

Thirty-eight native Mandarin speakers (15 males, mean age = 23.50 ± 2.61, range = 19–29 years) with normal or corrected-to-normal vision participated in the experiment, and they were paid for their participation. We used the software G^*^Power 3.1 (Faul et al., 2009) to conduct the power analysis, which revealed that a sample size of 34 participants would be required to detect an effect size (dz) of 0.5 with an α of 0.05 and power of 0.80. Ethics approval for the study was obtained from the ethics review board of Department of Psychology, Renmin University of China (the approval number 22-025). All participants gave written informed consent before the experimental session and received payment for their participation.

### 2.2 Materials

One hundred and twenty-nine black-and-white pictures with disyllabic names were selected from the database established by Zhang and Yang (2003). Among them, 120 pictures were used for the experiment and nine for practice sessions. The target trials comprised two types of scenarios, each consisting of four pictures. Participants were asked to describe the scenarios using a fixed syntactic structure, which was a vague ellipsis sentence (i.e., the target sentence) with two potential interpretations when presented visually. This is illustrated in the following examples:

Target sentence: “乌龟在青蛙的上面,不是孔雀。” (in English, “The turtle is above the frog, not the peacock.”).

One interpretation: “乌龟在青蛙的上面,不是孔雀在青蛙的上面。” (in English, “The turtle is above the frog, not the peacock is above the frog.”).

The other interpretation: “乌龟在青蛙的上面,不是乌龟在孔雀的上面。” (in English, “The turtle is above the frog, not the turtle is above the peacock.”).

In both interpretations, the first clause serves as the positive statement (e.g., “乌龟在青蛙的上面”, in English: “The turtle is above the frog”), and the second clause functions at the negative statement (e.g., “不是孔雀在青蛙的上面,” in English: “not the peacock is above the frog”). The key distinction between the positive and negative clauses lies in the focus (e.g., “乌龟,” in English: “the turtle”). Each type of scenarios corresponds to one interpretation, corresponding to initial and medial focus, respectively (see Tables 1a, b). Participants would accentuate the focus in the target sentence to accurately describe the scenario, which only represents one interpretation (Burght et al., 2021; Winkler, 2019). There are two positions of focus with accentuation (initial vs. medial, hereinafter “initial focus accentuation” and “medial focus accentuation” respectively).

**Table 1:** Example of three type of scenarios.

**Position of focus with accentuation**	**Scenario**	**Sentences**
Initial	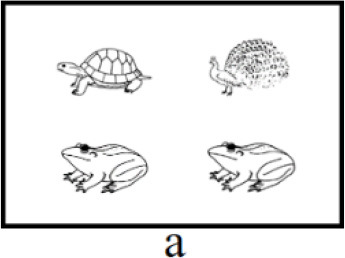	乌龟在青蛙的上面,不是孔雀。(a) The turtle is above the frog, not the peacock.
Medial	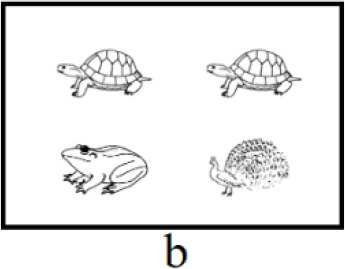	乌龟在青蛙的上面,不是孔雀。(b) The turtle is above the frog, not the peacock.
End of positive aspect (filler)	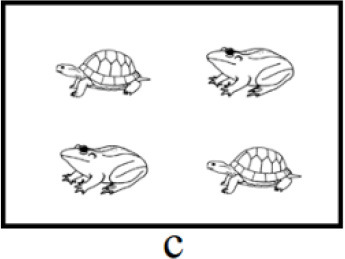	乌龟在青蛙的上面,不是下面。(c) The turtle is above the frog, not below.

Underlined is the focus with accentuation. Initial, initial position; Medial, medial position.

The left and right parts of each scenario can represent the positive and negative aspects of the sentence. Prior to each block, participants will receive information regarding the position of the positive aspect (left or right) within the scenarios and the orientation of the description (“above” or “below”) in the target sentence. In Table 1, the positive aspect in all scenarios was positioned on the left and the description orientation was “above.” Participants used the same sentence to describe scenarios a or b, albeit with different positions of focus with accentuation. The sentence describing scenario a is “乌龟在青蛙的上面,不是孔雀” (in English: “The turtle is above the frog, not the peacock,” featuring initial focus accentuation. The underlined is the focus with accentuation). This sentence conveys “the turtle is above the frog, not the peacock is above the frog.” The sentence describing scenario b is “乌龟在青蛙的上面,不是孔雀” (in English: “The turtle is above the frog, not the peacock.”), with medial focus accentuation, conveying “the turtle is above the frog, not the turtle is above the peacock.”

For filler trials, in order to prevent participants from anticipating only two positions of focus with accentuation (initial, medial), a third type of scenario was introduced (see Table 1c). Participants described scenario c using the sentence “乌龟在青蛙的上面,不是下面” (in English: “The turtle is above the frog, not below”), with focus accentuation at the final position of the positive aspect.

Among the 120 pictures, 24 were of animals, while the remaining 96 depicted non-animal objects, including artifacts, body organ, natural phenomena and foods. A scenario consists of four object pictures, and two of them are identical. Thus, 120 experimental pictures were divided into 40 sets. To prevent potential confounding due to differences in animacy among pictures, the three pictures within a scenario are selected exclusively from either animal or non-animal pictures. To ensure consistency across experimental conditions and avoid potential differences arising from varying experimental materials, both initial and medial accentuation conditions utilized the same set of pictures. As shown in scenarios a and b, the combination of “turtle,” “frog” and “peacock” appeared in both conditions. Additionally, to avoid the potential confounding stemming from differences in semantic categories between accented and deaccented pictures within a sentence, we interchanged the pictures in the positive aspect of a set to generate new scenarios. Notably, the tones of the keywords (i.e., the picture names in the positive aspect) within each set were not entirely uniform. The procedure described above ensured that the tones of the keywords at critical positions (i.e., initial and medial) were evenly distributed across conditions, thereby reducing the impact of tone on the results. The arrangement of three pictures within each set generated 6 scenarios, including 2 fillers and 4 experimental trials. With a total of 40 sets employed in the current study, the overall number scenarios amounted to 240. Furthermore, the positions of accented and deaccented pictures on the screen were evenly distributed, ensuring an equal frequency of appearances across four screen locations.

### 2.3 Design

The experiment was a single factor design with two levels (position of focus with accentuation: initial and medial focus accentuation). Each level comprised of 80 trials, resulting in 160 target trials. The total experiment consisted of 240 trials, including 160 targets and 80 fillers. The trials were subdivided into 8 blocks, with each containing 30 trials: 20 targets and 10 fillers. Within each block, the position of the positive aspect and the description orientation were consistent. Across the blocks, two versions of the experiment were administered: In the first version, the position of positive aspect alternated between the left and right sides, and the description orientation followed a balanced “ABBABAAB” pattern (where A represented “above” and B represented “below”). In the second version, the position of positive aspect was consistent with s that of version 1, but the description direction was reversed. Each participant was assigned only one version of the experiment. A break was provided between two blocks to ensure participant comfort and to minimize fatigue.

### 2.4 Procedures and apparatus

Before the main experiment, participants were instructed to familiarize themselves with all pictures and associated names by viewing them on a computer screen. Following this, participants underwent the practice trials for familiarizing themselves with experimental procedures. They were then asked to comfortably position their heads on the bracket, with the task involving describing scenarios using fixed ellipsis sentence. The stimuli were presented on a 19-inch DELL monitor with a resolution of 1920 × 1,080 pixels and a refresh rate of 60 Hz). Participants were seated 70 cm away from the monitor. Speech responses were recorded via a microphone connected to the YAMAHA Steinberg CI1 (Germany). Presentation of stimulus presentation was managed using Experiment Builder 2.3.1 software. Eye movements were tracked using an Eyelink Portable Duo eye tracker (SR Research, Canada) with a sampling rate of 1,000 Hz.

To improve the probability of participants accurately accentuating focus information, they were informed beforehand that their produced sentences would be recorded and played to others, who were required to select the scenario seen by participants among multiple scenarios. Prior to each block, the eye tracker was calibrated to the screen using a built-in 9-point calibration protocol. The eye tracker was recalibrated when the calibration accuracy exceeded a mean threshold of 0.5° and a maximum threshold of 1° visual angle. Subsequently, the instruction regarding the position of positive aspect in scenarios and the describe orientation were presented in the middle of the screen. The experimental session proceeded until participants could accurately accentuate the focus.

**Figure 1:**
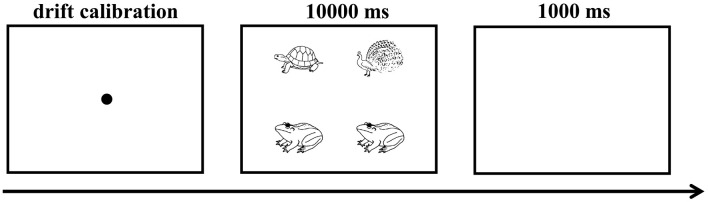
Visualization of the experimental trial.

During both the practice and experimental sessions, each trial involved the following sequences. Initially, a drift calibration to the center (black dots located at 960, 540) was presented, which was followed by the target scenario with each picture having a resolution of 200 × 200 pixels. The upper left picture was positioned at 560, 300, the upper right picture at 1,360, 300, the lower left picture at 560, 780, and the lower right picture at 1,360, 780. Participants were asked to describe the scenarios as accurately as possible. The scenario remained on the computer screen while participants were speaking. However, if participants did not utter any speech within 10 s after the scenario onset, it would disappear automatically. Participants were then requited to press the space bar to indicate that they had finished speaking. The next trial was started after a 1,000 ms blank screen (see Figure 1).

## 3 Results

All incorrect (where participants named pictures incorrectly), disfluent responses and data beyond three standard deviations of the mean value in the speech latency (5.28% of original data) were excluded from the acoustic, speech latency, and the eye-tracking analyses.

### 3.1 Acoustic analyses of accentuation

We conducted acoustic analyses to confirm that participants spontaneously accented the focus. Praat software was used to segment the sentences generated by each subject into seven segments. Below is an example illustrating the segmentation, with the symbol | indicating the position of audio segmentation:

乌龟|在|青蛙|的|上面|,|不是|孔雀。

(In English: The turtle | is | above | the frog |, | not | the peacock.)

(Pinyin: Wu1gui1 | zai4 | qing1wa1 | de | shang4mian4 |, | bu2shi4 | kong2que4.)

**Figure 2:**
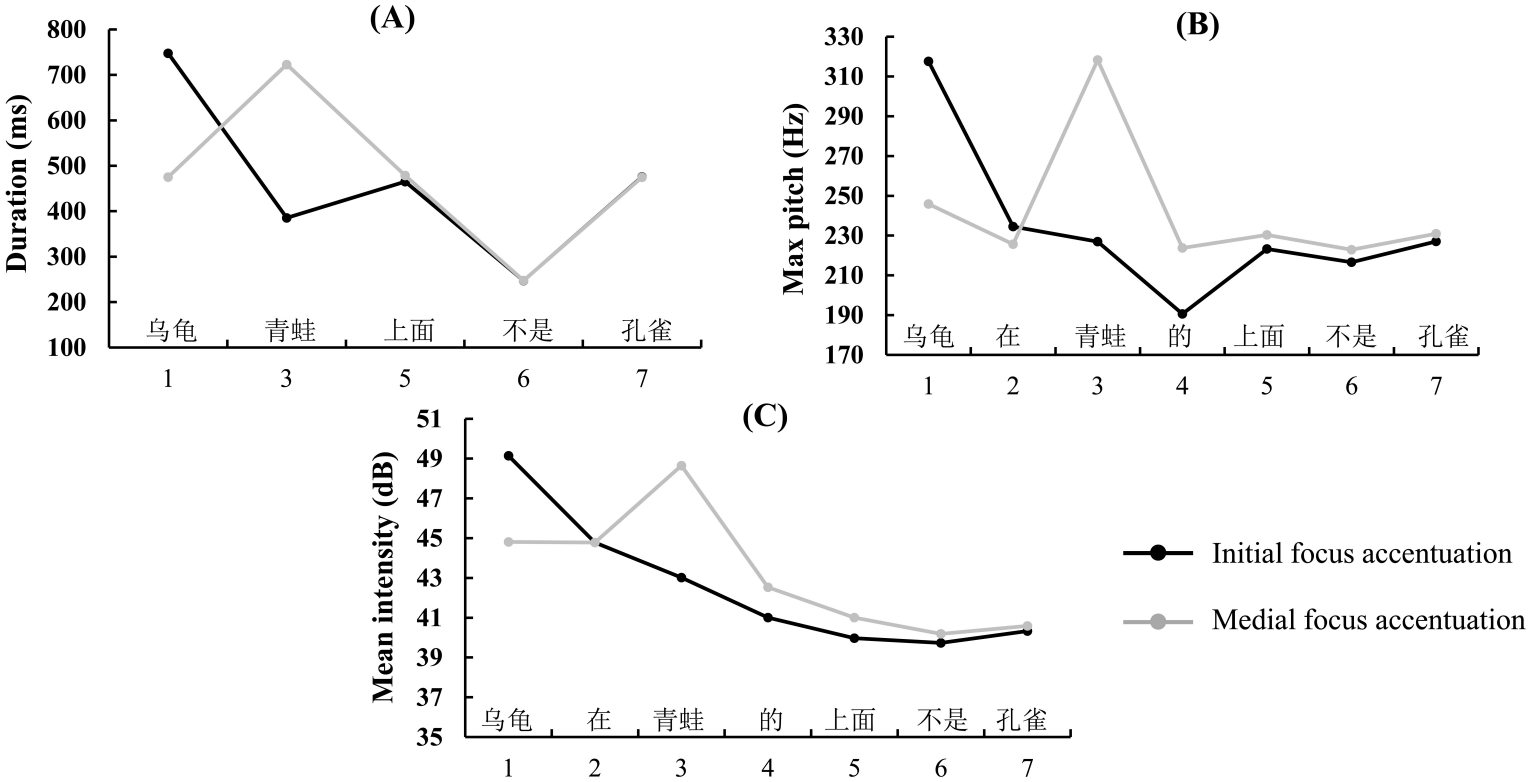
Mean measurements of the **(A)** duration, **(B)** max pitch, and **(C)** mean intensity of each sentence segment, broken down by positions of focus with accentuation. The abscissa is the segment number. For example, in “乌龟|在|青蛙|的|上面|, |不是|孔雀”, segment 1 is “乌龟”, segment 2 is “在”, segment 3 is “青蛙”, segment 4 is “的”, segment 5 is “上面”, segment 6 is “不是”, segment 7 is “孔雀”. Note that only durations of segment 1, 3, 5, 6, and 7, which have the same number of syllables, were compared.

The script developed by Xu (1999) was used to extract the duration (in milliseconds), maximum pitch (in hertz), and mean intensity (in decibels) for each audio segment. With the exception of fillers, each acoustic parameter of the focused segment was compared with other non-focused segments. When a spoken sentence where the acoustic parameters of the intended focus segment were significantly higher than those of the other six segments, indicating that the focus segment was accented successfully. The average proportion of such trials in all participants was 92.00% (SD = 7.92%), indicating that the participants spontaneously accented the focus in most trials. Paired sample *t*-tests were performed on the acoustic data from correctly accentuated trials, comparing the focused segments to non-focused segments. As the number of syllables varied across segments, duration analysis was performed on segments with equal syllable counts (i.e., segments 1, 3, 5, 6, and 7). Results showed that the duration, maximum pitch, and mean intensity of the focused segments were significantly greater than those of the other segments (*ps* < 0.001) (see Table 2), indicating that participants accented the focus as expected. The results of the acoustic parameter are illustrated in Figure 2.

**Table 2:** Acoustic parameters and *t*-test results for the focused and non-focused segments in sentences with initial and medial focus.

**Acoustic parameter**	**Initial accentuation**							**Medial accentuation**						
	**Focused segment**	***M*** **(SD)**	**Non-focused segment**	***M*** **(SD)**	***df***	***t***	**Cohen's D**	**Focused segment**	***M*** **(** ***SD*** **)**	**Non-focused segment**	***M*** **(SD)**	***df***	***t***	**Cohen's D**
Duration (ms)	1	747.55 (109.49)	3	385.38 (52.31)	37	18.70^***^	3.82	3	722.95 (124.58)	1	474.96 (73.61)	37	12.48^***^	2.29
			5	465.26 (67.59)	37	14.80^***^	2.95			5	478.54 (67.02)	37	12.46^***^	2.26
			6	247.33 (45.77)	37	29.94^***^	5.25			6	247.42 (46.63)	37	23.41^***^	4.36
			7	475.54 (49.95)	37	15.20^***^	2.87			7	474.56 (54.40)	37	12.40^***^	2.30
Max pitch (Hz)	1	317.61 (65.21)	2	234.49 (59.30)	37	19.98^***^	1.33	3	318.32 (69.80)	1	245.84 (65.77)	37	13.80^***^	1.07
			3	226.95 (62.35)	37	14.64^***^	1.42			2	225.69 (62.45)	37	20.38^***^	1.39
			4	190.61 (50.73)	37	22.86^***^	2.14			4	223.71 (53.22)	37	21.60^***^	1.50
			5	223.27 (54.15)	37	17.06^***^	1.56			5	230.33 (58.66)	37	18.62^***^	1.35
			6	216.63 (59.92)	37	19.93^***^	1.61			6	222.88 (66.93)	37	17.99^***^	1.40
			7	227.06 (56.79)	37	17.41^***^	1.47			7	230.90 (61.83)	37	16.11^***^	1.32
Mean intensity (dB)	1	49.15 (3.34)	2	44.78 (3.68)	37	12.40^***^	1.24	3	48.65 (3.50)	1	44.81 (3.00)	37	9.42^***^	1.17
			3	43.02 (3.18)	37	15.73^***^	1.88			2	44.78 (3.40)	37	8.55^***^	1.12
			4	41.00 (3.73)	37	14.38^***^	2.30			4	42.53 (4.49)	37	10.66^***^	1.50
			5	39.97 (3.62)	37	17.88^***^	2.63			5	41.01 (3.62)	37	18.19^***^	2.15
			6	39.73 (2.98)	37	20.25^***^	2.97			6	40.19 (2.96)	37	16.47^***^	2.59
			7	40.33 (3.08)	37	17.83^***^	2.74			7	40.58 (3.08)	37	16.07^***^	2.44

Each sentence was divided into seven segments, such as “乌龟|在|青蛙|的|上面|,|不是|孔雀” (meaning “The turtle is above the frog, not the peacock.” Pinyin version: Wu1gui1 | zai4 | qing1wa1 | de | shang4mian4 |, | bu2shi4 | kong2que4; English version: The turtle | is | above | the frog |, | not | the peacock. The symbol | indicates the audio segmentation position.). Segment 1 is “乌龟”; Segment 2 is “在”; Segment 3 is “青蛙”; Segment 4 is “的”; Segment 5 is “上面”; Segment 6 is “不是”; Segment 7 is “孔雀.” The standard deviation is displayed in brackets. ^***^p < 0.001.

### 3.2 Speech latencies

**Figure 3:**
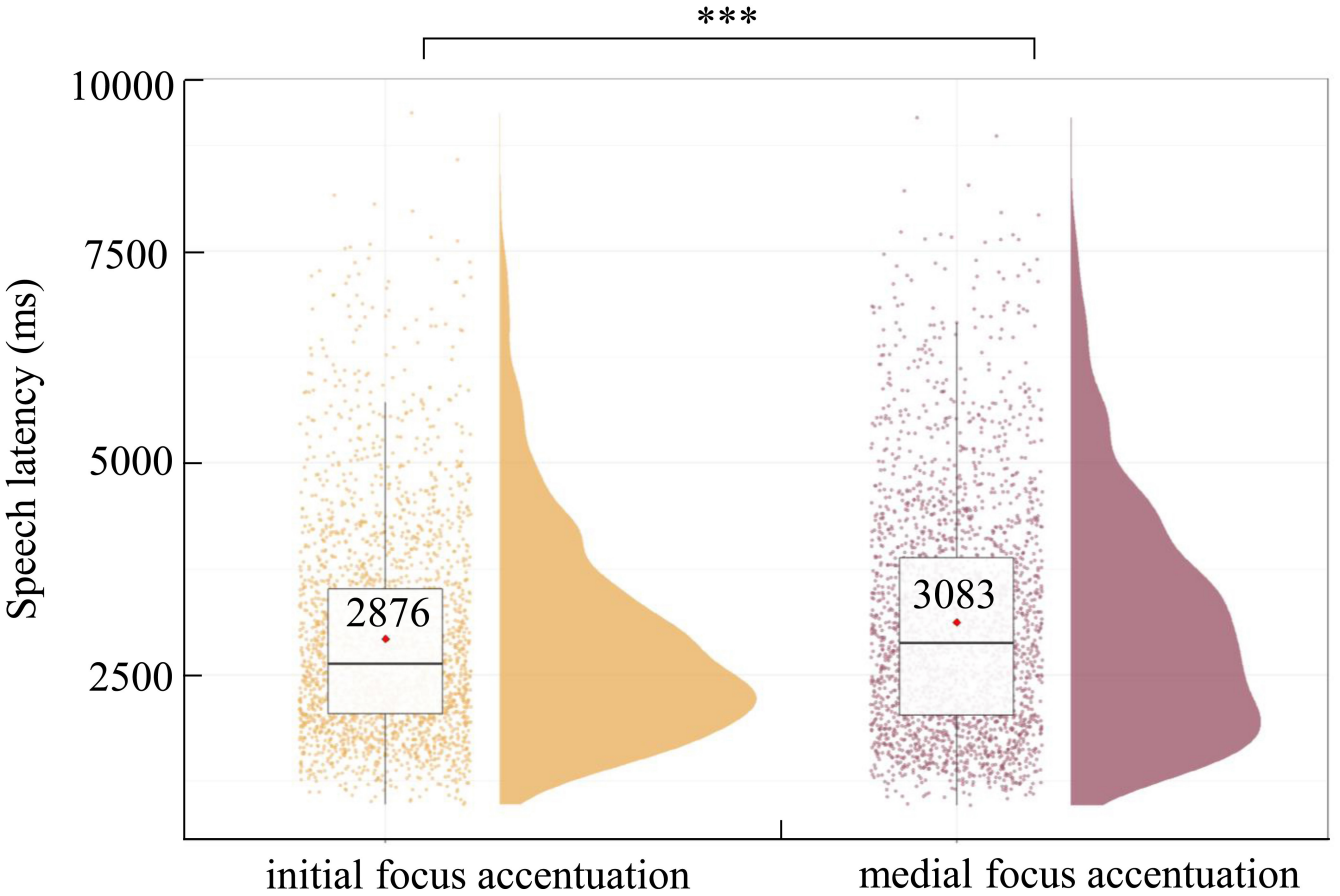
Speech latencies in sentence with initial **(left)** and medial **(right)** focus accentuation. The thin horizontal black line represents the median. The violin plot outline shows the density of data points for different dependent variables, and the boxplot shows the interquartile range with the 95% confidence interval represented by the thin vertical black line. The red diamonds in the boxplot denote the mean per condition, above which is written the mean value. The full dots represent individual data points. ***p < 0.001.

Speech latencies refer to the duration between the onset of scenario presentation and when participants start speaking. Figure 3 shows the latencies of spoken sentences with two positions of focus with accentuation (initial vs. medial). These speech latencies were analyzed using a linear mixed-effects model (LMM) analysis with the package lmerTest (Kuznetsova et al., 2017) in R (version 4.1.2). We initially constructed a model including position of focus with accentuation as a fixed effect, picture position as a covariate, and random intercepts for participants and items with by-participant as well as by-item random slopes for position of focus with accentuation (Baayen et al., 2008). However, this fully specified random effects structure failed to converge, we removed random slopes for items, given that in researcher-designed experiments, the variance for items typically tends to be smaller than that for participants (Segaert et al., 2016), leading to convergence. The best-fitting model included position of focus with accentuation as a fixed effect, picture position as a covariate, random intercepts for participants and items, and by-participant random slopes for position of focus with accentuation. Result showed that speech latencies in sentences with medial focus accentuation were significantly longer than those in sentences with initial focus accentuation (β = 206.38, *p* = 0.001). To assess the reliability of the main effect of position of focus with accentuation, we performed a Bayesian factor analysis using the lmBF program from the BayesFactor package (Morey et al., 2022) in R software, and the results revealed that the Bayes factor of BF_10_ was 79.60, providing a robust support for the main effect of position of focus accentuation (Jeffreys, 1961) (see Figure 3 and Table 3).

**Table 3:** Fixed effects of a linear mixed effects model with speech latencies as the dependent variable.

**Predictor**	**β**	***SE***	***df***	***t***	***p***
Intercept	2970.61	133.23	53.26	22.30	<0.001
Picture position: Lower left vs. Upper left	4.88	74.03	153.87	0.07	0.947
Picture position: Upper right vs. Upper left	−183.24	74.14	154.73	−2.47	0.015
Picture position: Lower right vs. Upper left	−198.16	73.88	152.47	-−2.68	0.008
Position of focus with accentuation: Medial vs. Initial	206.38	62.98	95.99	3.28	0.001

Initial, initial position; Medial, medial position.

### 3.3 Proportion of fixation duration before articulation

Proportion of fixation duration refers to the ratio of total fixation time for a specific ROI to the total fixation time within the period of interest. In each scenario, the positive aspect consisted of two ROIs: one defined as the accented picture, and the other as the deaccented picture. The period on interest spanned from the onset of the scenario to the onset of speech. To compare the accented and deaccented segments during the interested period, trials where participants fixated solely on one picture were excluded from all analyses. Among the 38 participants, the percentage of trials wherein only one picture was fixated upon before articulation ranged from a minimum of 0.00% and a maximum of 33.13% (*M* = 9.13%, SD = 8.19%). There were 2,406 trials involving sentences with initial focus accentuation and 2,386 trials involving sentences with medial focus accentuation.

**Figure 4:**
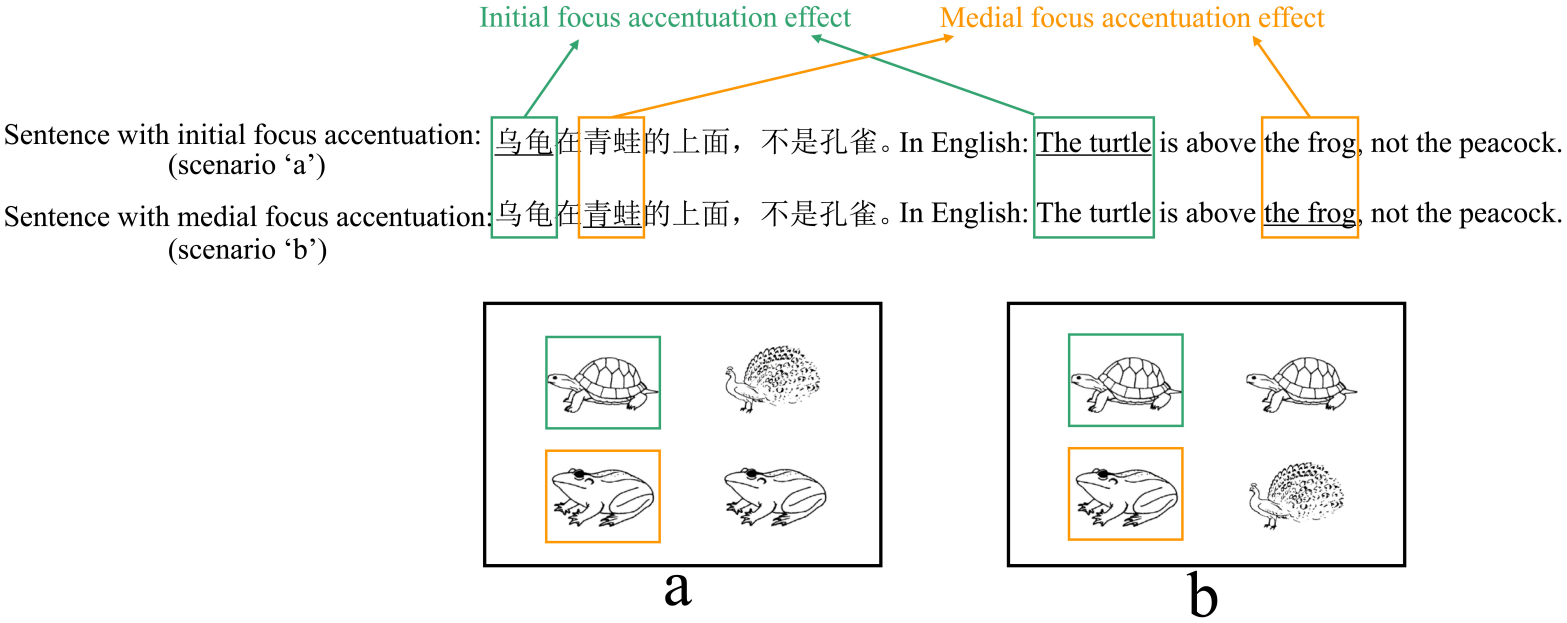
Focus accentuation effect at initial and medial position in eye movement analysis. The positive part in all scenarios were on the left and the orientation of description were “above.” Underlined is the focus with accentuation. Green for initial focus accentuation effect. Yellow for medial focus accentuation effect.

To investigate the presence of a focus accentuation effect in different position, we compared the proportion of fixation duration on accented and deaccented picture within the same sentence positions (see Figure 4). Similar to analyses conducted by Ganushchak et al. (2014, 2017), we transformed the single factor design with two levels (position of focus with accentuation: initial and medial focus accentuation) into a 2 × 2 design, incorporating word position (initial, medial) and accented state (accented, deaccented) as factors for LMM analysis. Fixed effects included accented state (accented, deaccented), word position (initial, medial), and their interaction. The picture position was added as a covariate, and random effects included random intercepts for participants and items, by-participant as well as by-item random slopes for accented state, word position, and their interaction. When the model did not converge with the maximal random effects structure, we simplified the random slopes, first removing the interactions and then removing the main effects in the order of least variance explained until the model converged (Barr et al., 2013).

**Figure 5:**
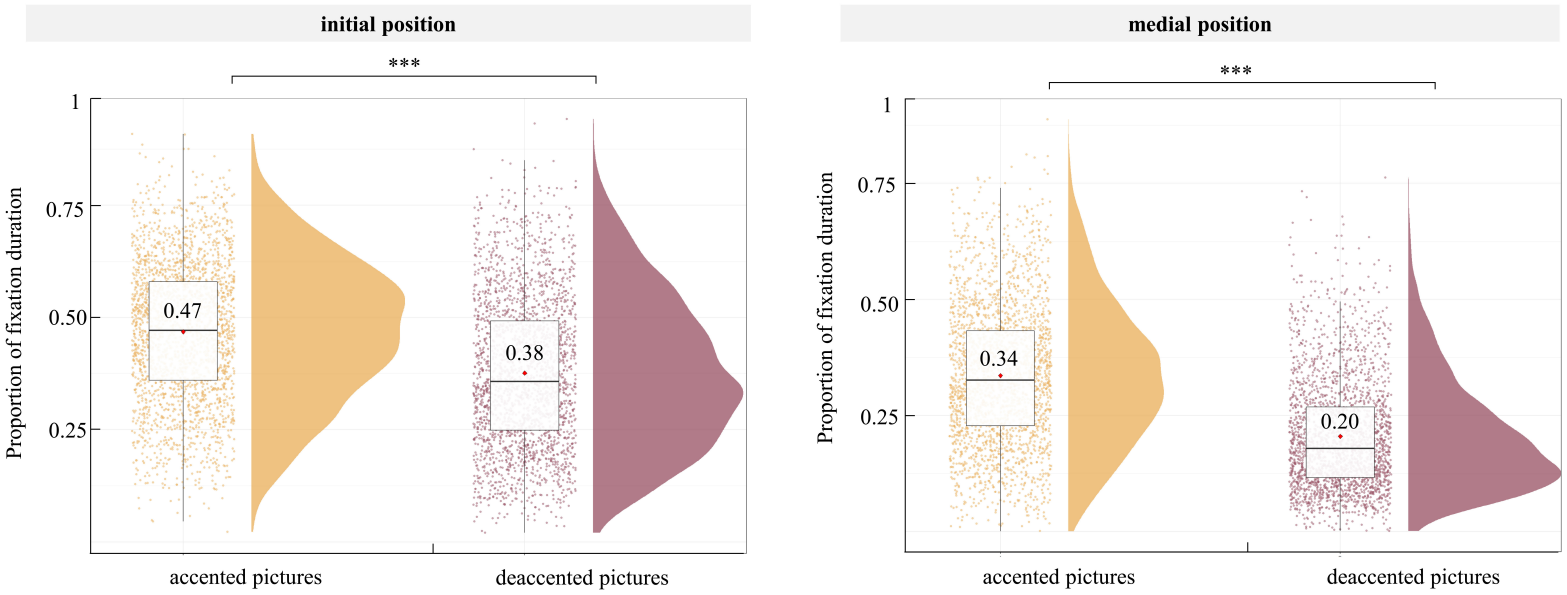
Proportion of fixation duration in sentence with initial **(left)** and medial **(right)** positions, across the accented state before articulation. The thin horizontal black line represents the median. The violin plot outline shows the density of data points for different dependent variables, and the boxplot shows the interquartile range with the 95% confidence interval represented by the thin vertical black line. The red diamonds in the boxplot denote the mean per condition, above which is written the mean value. The full dots represent individual data points. ***p < 0.001.

The best-fitting model included accented state, word position, and their interaction as fixed effects, the picture position as a covariate. Random intercepts for participants and items, by-participant random slopes for accented state, word position, and their interaction, as well as by-item random slopes for accented state were included as random effects. The best fitting model did not differ significantly from the full model (full model: Akaike information criterion [AIC] = −12013, Bayesian information criterion [BIC] = −11812; best-fitting model: AIC = −12019, BIC = −11869, *p* = 0.343). The results showed an interaction between accented state and word position is significant (β = −0.04, *p* < 0.001). Further simple effects analysis showed that in two positions, the proportion of fixation duration on accented pictures was significantly higher than that on deaccented pictures (initial position: β = 0.09, *p* < 0.001; medial position: β = 0.13, *p* < 0.001) (see Table 4 and Figure 5). To assess the reliability of this interaction, we performed a Bayesian factor analysis, and the results showed that the Bayes factor of BF_10_ was 806.74, providing an extremely robust support for the interaction.

**Table 4:** Simple effects of the interaction between accented state and word position with the proportion of fixation duration as the dependent variable before articulation.

**Word position**	**Contrast**	**β**	***SE***	***z***	***p***
Initial position	Accented vs. Deaccented	0.09	0.01	9.07	<0.001
Medial position	Accented vs. Deaccented	0.13	0.01	14.12	<0.001

Accented, accented pictures; Deaccented, deaccented pictures.

### 3.4 Time-course of speech planning before articulation

To explore how focus and accentuation are encoded in speech production, we conducted the time-course analysis by comparing fixation patterns between accented and deaccented pictures in two distinct approaches.

**Figure 6:**
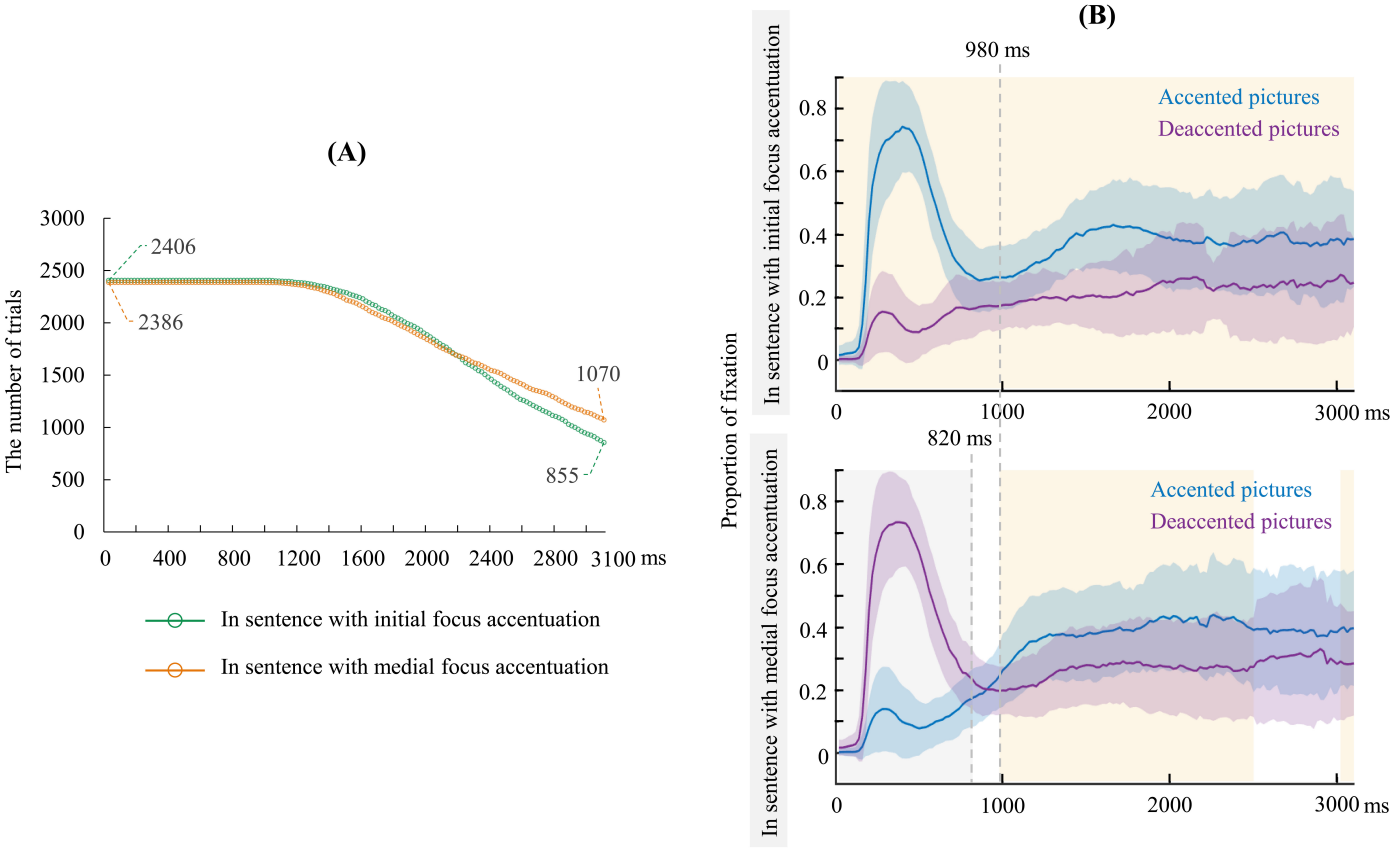
**(A)** The number of trials in the time-course analyses in sentence with initial and medial focus accentuation before articulation. **(B)** The proportion of fixation on accented and deaccented pictures in sentences with an initial (upper) and a medial (lower) focus accentuation before articulation (0 ms indicates picture onset). The gray dashed lines were the time window boundaries for the analyses (820, 980 ms, respectively). The shadows were time windows with significant differences in the permutation test results (the yellow shadow: accented > deaccented; the gray shadow: accented < deaccented).

First, we compared the fixation patterns of accented and deaccented picture within the same sentence, separately for with initial and medial focus accentuation, in each time bin (20 ms), within the interested time interval from the scenarios onset to speeches onset (average 3,100 ms). As the time window shifted with a step size of 20 ms, the number of trials included in the analysis decreased accordingly. Figure 6A presents the number of trials included in each time bin, reflecting that the number of trials involved in the following analyses was sufficient to prove the validity of the subsequent results. Figure 6B plots the proportion of fixation on accented and deaccented pictures in sentences with initial and medial focus accentuation, respectively. Given the current uncertainty regarding the timing of various processes involved in sentence production research, we employed a data-driven approach to select the time windows for analysis. Specifically, we performed a non-parametric permutation test, and *p*-values were corrected by a false discovery rate (FDR) of 0.05 (Genovese et al., 2002). The results showed that sentences with initial focus accentuation had a significant difference between accented and deaccented pictures in the time window of 0–3100 ms, while sentences with medial focus accentuation had significant differences in the time windows of 0–820 ms, 980–2500 ms, and 3020–3100 ms (all *ps* < 0.05, FDR corrected) (see Figure 6B). Based on the results of permutation tests, we selected two time windows for each type of sentence. For sentences with initial focus accentuation, the chosen time windows were 0–980 ms and 980–3100 ms; while for sentences with medial focus accentuation, the chosen time windows were 0–820 ms, and 980–3100 ms.

The proportion of fixation as dependent variable was analyzed using LMM analysis. We started from a model including accented state (accented, deaccented) as a fixed effect, picture position as a covariate, random intercepts for participants and items, and by-participant as well as by-item random slopes for accented state. As this model did not converge, we removed random slopes for items and convergence was reached. The best-fitting model included accented state as a fixed effect, the picture position as a covariate, random intercepts for participants and items, and by-participant random slopes for accented state. This analysis was performed in each time window separately. Results of LMM analysis showed a significant main effect of accented state in each time window (see Table 5). For sentences with initial focus accentuations, the proportion of fixation on accented picture was significantly higher than that on deaccented picture in each time window (0–980 ms: β = 0.30, *p* < 0.001; 980–3100 ms: β = 0.15, *p* < 0.001). The results of Bayesian factor analysis showed that in 0–980 ms: BF_10_ > 100; in 980–3100 ms: BF_10_ > 100, showing an extreme support for the main effect of accented state. For sentences with medial focus accentuations, the proportion of fixation on accented picture was significantly lower than that on deaccented picture in 0–820 ms (β = −0.33, *p* < 0.001), while this pattern was reversed in 980–3100 ms (β = 0.15, *p* < 0.001). The results of Bayesian factor analysis showed that in 0–820 ms: BF_10_ > 100; in 980–3100 ms: BF_10_ > 100, showing an extreme support for the main effect of accented state.

**Table 5:** Fixed effects of a linear mixed effects model with the proportion of fixation as the dependent variable in sentence with initial and medial focus accentuation before articulation.

**Time window**	**Predictor**	**Sentence with initial focus accentuation**					**Sentence with medial focus accentuation**				
		β	***SE***	***df***	***t***	***p***	β	***SE***	***df***	***t***	***p***
0–980 ms	Intercept	0.45	0.01	57.08	42.95	<0.001					
	Picture position: Lower left vs. Upper left	−0.08	0.01	4662.00	−12.62	<0.001					
	Picture position: Upper right vs. Upper left	0.01	0.01	178.20	1.30	0.195					
	Picture position: Lower right vs. Upper left	−0.09	0.01	179.10	−12.24	<0.001					
	Accented state: Accented vs. Deaccented	0.30	0.01	37.02	21.67	<0.001					
0–820 ms	Intercept						0.14	0.01	52.30	14.00	<0.001
	Picture position: Lower left vs. Upper left						−0.08	0.01	4619.00	−13.22	<0.001
	Picture position: Upper right vs. Upper left						0.01	0.01	265.60	0.12	0.903
	Picture position: Lower right vs. Upper left						−0.01	0.01	266.00	−15.16	<0.001
	Accented state: Accented vs. Deaccented						−0.33	0.01	36.86	−20.33	<0.001
980–3100 ms	Intercept	0.38	0.01	43.49	22.94	<0.001	0.41	0.01	41.25	19.01	<0.001
	Picture position: Lower left vs. Upper left	−0.04	0.01	4737.00	−4.89	<0.001	−0.02	0.01	4694.00	−2.41	0.016
	Picture position: Upper right vs. Upper left	−0.01	0.01	4739.00	−0.41	0.682	0.01	0.01	4698.00	1.02	0.308
	Picture position: Lower right vs. Upper left	−0.03	0.01	4740.00	−4.61	<0.001	−0.01	0.01	4698.00	−1.79	0.073
	Accented state: Accented vs. Deaccented	0.15	0.01	36.98	5.42	<0.001	0.15	0.01	36.97	4.12	<0.001

**Figure 7:**
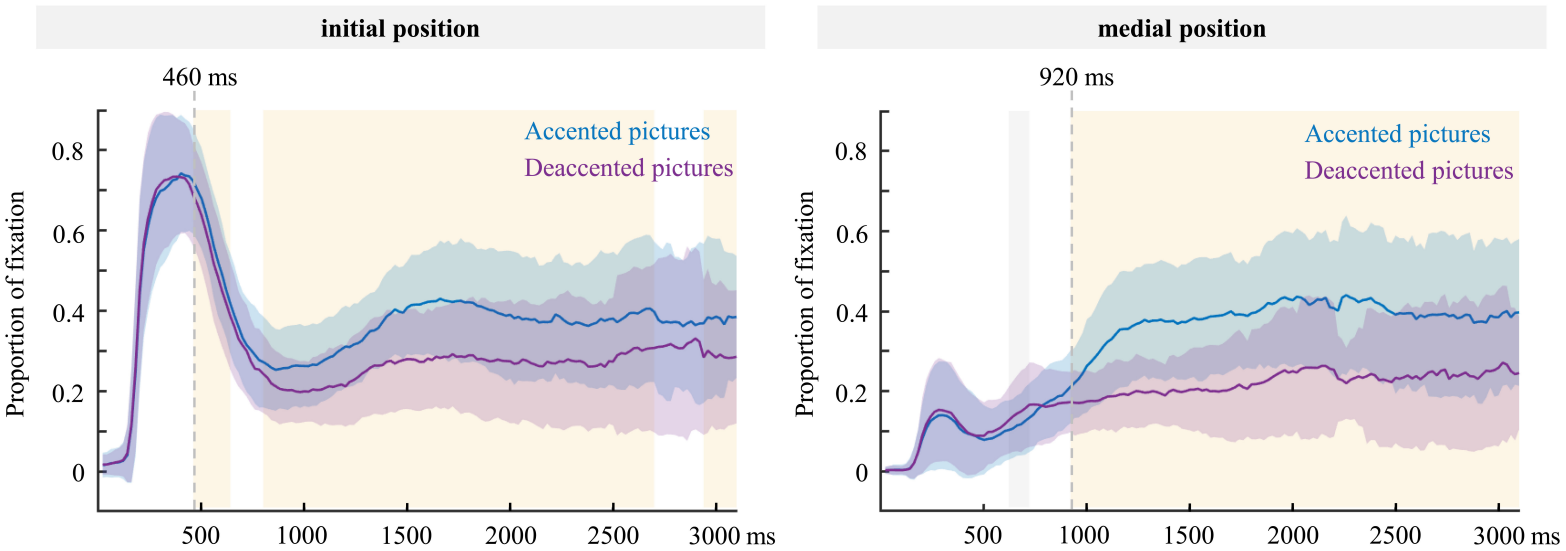
The proportion of fixation on accented and deaccented pictures in sentences with initial **(left)** and medial **(right)** positions before articulation (0 ms indicates picture onset). The gray dashed lines were the time window boundaries for the analyses (460, 920 ms respectively). The shadows were time windows with significant differences in the permutation test results (the yellow shadow: accented > deaccented; the gray shadow: accented < deaccented).

Second, we performed the same time-course analyses and permutation tests as above to compared the fixation patterns of accented and deaccented pictures in sentences with initial and medial positions, respectively, known as the accentuation effect. Figure 7 plots the proportion of fixation on accented and deaccented pictures in sentences with initial and medial positions, respectively. The results of permutation tests showed that in sentences with initial positions, the differences between accented and deaccented were significant in the time windows of 460–640 ms, 740–760 ms, 800–2700 ms and 2940–3100 ms, while in sentence with medial position, the differences between accented and deaccented pictures significant in the time windows of 620–720 ms and 920–3100 ms (all *ps* < 0.05, FDR corrected) (see Figure 7). We assumed that the processing of accentuation was reflected only when the proportion of fixation on accented picture was significantly greater than that on deaccented picture. Based on this assumption and the results of permutation tests, we selected 460–3100 ms for sentence with initial position and 920–3100 ms for sentence with medial position. A similar LMM analysis as above was performed.

The LMM analysis results showed a significant main effect for accented state in each time window (see Table 6). The proportion of fixation duration on accented picture was significantly higher than that on deaccented picture in each time window (In sentence initial position, 460–3100 ms: β = 0.09, *p* < 0.001. In sentence medial position, 920–3100 ms: β = 0.18, *p* < 0.001). The results of Bayesian factor analysis showed that in sentences with initial position (460–3100 ms): BF_10_ > 100; in sentences with medial position (920–3100 ms): BF_10_ > 100, providing an extreme support for the main effect of accented state.

**Table 6:** Fixed effects of a linear mixed effects model with the proportion of fixation as the dependent variable in sentence initial and medial position before articulation.

**Word position**	**Time window**	**Predictor**	**β**	***SE***	***df***	***t***	***p***
Initial position	460–3100 ms	Intercept	0.39	0.01	74.04	30.46	<0.001
		Picture position: Lower left vs. Upper left	−0.01	0.01	154.89	−1.47	0.143
		Picture position: Upper right vs. Upper left	−0.01	0.01	156.00	−0.48	0.634
		Picture position: Lower right vs. Upper left	−0.03	0.01	152.95	−3.44	<0.001
		Accented state: Accented vs. Deaccented	0.09	0.01	62.86	8.85	<0.001
Medial position	920–3100 ms	Intercept	0.41	0.02	47.23	18.96	<0.001
		Picture position: Lower left vs. Upper left	−0.03	0.01	143.07	−2.99	0.003
		Picture position: Upper right vs. Upper left	0.01	0.01	131.53	0.61	0.546
		Picture position: Lower right vs. Upper left	−0.01	0.01	139.97	−1.52	0.131
		Accented state: Accented vs. Deaccented	0.18	0.01	51.15	15.18	<0.001

## 4 Discussion

To explore how conceptualization and prosodic encoding proceeds in speech production, we investigated which incremental framework (linear incrementality and hierarchical incrementality) is followed for focus and accentuation processing during real-time speech production. Utilizing the visual world eye-tracking paradigm, we investigated the production of sentences with varied positions of focus with accentuation. The findings indicated that the position of focus with accentuation influences sentences planning.

Behavioral results showed that speech latencies in sentences with initial focus accentuation were significantly shorter than in sentences with medial focus accentuation, indicating that compared with sentences with initial focus accentuation, sentences with medial focus accentuation entail a greater cognitive load during pre-articulatory planning. This increased load may stem from a broader planning scope and more extensive planning content, such as the encoding of focus with accentuation in the medial of sentences as well as preceding information. The planning scopes of initial and medial focus accentuation sentences are distinct for the following reasons. If the planning scope of sentences with initial and medial focus accentuation were the same, there would be two possible cases, one is the planning scope is small, possibly the first prosodic word (e.g., Meyer, 1996; Schriefers, 1999; Jescheniak et al., 2003; Wheeldon and Lahiri, 1997), and the other is the planning scope is large, possibly the entire sentence (e.g., Garrett, 1975, 1976; Ferreira, 1993). If it is the first case, it seems plausible that speakers process both the segmental properties and accentuation information of the first prosodic word. Compare to initial focus accentuation sentences, the first prosodic word of medial focus accentuation sentences is deaccented, therefore it takes less planning early on, resulting in longer speech latencies for initial accentuation sentences. If it is the second case, because initial and medial focus accentuation sentences are identical in sentence structure and segment content, differing only in position of focus with accentuation, there may be no significant difference in their speech latencies, as we predict for hierarchical incrementality. However, we found that the speech latencies of medial focus accentuation sentences were longer than initial focus accentuation sentences, indicating that the planning scope of these sentences were different and the processing of focus and accentuation is not an overall process, but in the order of occurrence, which supports linear incrementality.

The behavioral finding aligns with research on stress encoding, in which it was found that speech latencies in the initial stressed word were significantly shorter than in the final stressed word (Schiller et al., 2004). This suggests that although accentuation and stress operate at different prosodic levels (sentences or words), they are processed similarly, following a pattern of linear incrementality. This finding was opposite with the findings of Ganushchak et al. (2014) regarding focus sentence production, where sentences with focus at the end were produced significantly faster than those with focus at the initial position. This discrepancy can be primarily attributed to the question–answer pairs experimental design employed in their study, which allowed participants to preview the initial information of answers in the question in sentences with focus at the end (e.g., “What is the policeman stopping?”), but not in sentences with focus at the initial position (e.g., “Who is stopping the truck?”). Despite the answers were the same across conditions (e.g., “The policeman is stopping the truck”), the preview of the initial information of the sentence had a significant facilitating effect on speech latency (Allum and Wheeldon, 2009). Consequently, this previewing facilitated the production of sentences with focus at the end.

The eye-tracking data substantiated our hypothesis regarding the differences in speech latency, indicating that the delay in producing sentence with focus accentuation in the medial position compared to those in the initial position arises because speakers encode not only the focus and accentuation in the medial position but also the preceding information. First, the results showed that for both initial and medial positions of sentences, the proportion of fixation duration on the accented picture was significantly higher than that on the deaccented picture. This indicates the presence of the focus accentuation effect, indicating that speakers encoded the focus accentuation at both sentence positions (initial, medial) before articulation. This finding aligns with the study by Ganushchak et al. (2014), who found that regardless of whether the focus in a sentence was at the beginning or end, speakers would encode focus information before articulation. Specifically, they noted more frequent and longer gazes at the focus picture when producing a subject-focused or object-focused sentence compared to a neutral-focused one, occurring 400 ms after the scenario presentation.

More importantly, the time-course analysis found that participants gave priority to the initial information of sentence when producing sentences with initial or medial focus accentuation. Specifically, when the scenario was presented for 980 ms, the proportion of fixations on the accented pictures was significantly greater than the deaccented ones in sentences with initial focus accentuations, whereas the reverse pattern emerged in sentences with medial focus accentuations. In previous eye-tracking research (Ganushchak et al., 2014; Griffin and Bock, 2000; Konopka et al., 2018; Schlenter et al., 2022; van de Velde et al., 2014; Gleitman et al., 2007; Konopka and Meyer, 2014), it has been assumed that the period within 400 ms of scenario presentation represents the preverbal message encoding stage (i.e., conceptualization), followed by a longer phase of linguistic encoding (after around 400 ms). Based on these assumptions, our results suggest that the initial increment of conceptualization and linguistic encoding consist only of information specific to one entity, and this entity predicts selection of starting points. This finding supports the notion of a linear incremental framework for sentence planning (Ganushchak et al., 2017; Gleitman et al., 2007; Schlenter et al., 2022). Furthermore, we found that even after 980 ms of scenario presentation, speakers maintained a preference for fixating on the initial information when producing sentences with initial focus accentuation. However, when producing sentences with medial focus accentuation, speakers shift their fixation preference toward the medial information (i.e., accented pictures), suggesting that the medial accentuation was processed prior to articulation. These eye-tracking findings support our speculation based on the results of speech latency. Unlike sentences with initial focus accentuation, producing sentences with medial focus accentuation entail a larger planning scope. Speakers not only need to encode the focus and accentuation in the middle before articulation but also encode preceding information when producing sentences with medial focus accentuation.

However, we are still unable to determine whether the encoding of initial accentuation precedes that of medial accentuation. To provide more direct evidence, we compared the onset times of focus accentuation effects in different positions. This comparison effectively controls for potential differences in the pronunciation difficulty of initial versus medial accentuation. Given that the syntactic structure and words were identical between sentences, differing only in focus and accentuation information, we assumed that focus accentuation effects reflect focus accentuation encoding. The results showed that at the sentence-initial position, the proportion of fixations on the accented pictures was significantly greater than the deaccented ones (i.e., the focus accentuation effect), starting from 460 ms after the scenario onset and lasting until articulation. Compared with the initial focus accentuation effect, the medial focus accentuation effect emerged later around 920 ms after the scenario onset, and persisted until before articulation. By integrating assumptions from previous eye-tracking studies (i.e., pre-400 ms after scenario onset is preverbal message encoding, and post-400 ms is linguistic encoding; see Ganushchak et al., 2014; Griffin and Bock, 2000; Schlenter et al., 2022; Gleitman et al., 2007; Konopka and Meyer, 2014) with our perspective (i.e., accentuation effects reflect accentuation encoding), we propose that in the initial position, the period from 0 ms (scenario onset) to 460 ms involves preverbal message encoding (including identification of the initial focus) and early linguistic encoding for the initial information, with accentuation encoding occurring after 460 ms.

For the medial position, we speculate that the period from 0 to 920 ms represents preverbal message encoding (including identification of the medial focus) and early linguistic encoding for the medial information, followed by accentuation encoding after 920 ms. As previously mentioned, in this study, speakers employed a strategy of linear incrementality in sentence planning, encoding concept by concept and word by word in the order of mention, hence the delayed onset of accentuation encoding for the medial information. This is consistent with behavioral results, suggesting that speakers encode the medial accentuation later than the initial accentuation due to they need more planning before articulation. It further supported the idea that when producing sentence with focus accentuation, instead of directly establishing a whole conceptual framework and a corresponding sentence-level accentuation pattern, speakers process focus and accentuation in a rightward incremental manner. In line with Levelt's (1989) view, he proposed that accentuation encoding can easily proceed incrementally, as speakers only need to ensure that the currently encoded focus content is more prominent in prosody than the previously encoded words, with no further emphasis on subsequent words. This is also the reason for the prolonged duration of accentuation encoding observed in this study.

Different from the linear incrementality strategy supported by our study, Ganushchak et al. (2014) found that regardless of whether the focus was at the beginning or end of the sentence, participants encoded focus information after 400 ms of scenario onset. Their finding supports the hierarchical incrementality framework, rather than the linear incrementality. Participants likely formed a whole conceptual framework containing focus information at different positions, enabling them to prioritize encoding focus information during linguistic encoding. This indicates that speakers do not consistently adopt a fixed framework to produce sentences with focus, instead, they begin formulation by prioritizing encoding of information that is easy to process (Konopka and Meyer, 2014). It is important to note that the experimental setup in Ganushchak et al. (2014) differs from that of the current study. In the study by Ganushchak et al. (2014), participants were required to produce a sentence with focus after hearing a question. Notably, the questions provided in the focus conditions (e.g., “Who is stopping the truck?”) contained nearly all the elements required for participants to construct the sentence, including sentence structure, verb and one noun, with only one noun left unmentioned. Consequently, participants were already primed with preverbal information about the relational structure of the event (i.e., event gist, one character stopping one thing) and one of the entities (a truck) in advance. Moreover, they also activated specific syntactic structure and lexical information of one of the nouns and the action, in which the activation of event gist and syntactic structure would facilitate participants in forming a whole conceptual framework. Previous study has demonstrated that the higher-codability of event gist and the priming of syntactic structure would increase the likelihood of early encoding of all information in a scenario, reflecting the generation of a complete conceptual framework (Konopka and Meyer, 2014; Konopka and Kuchinsky, 2015; Kuchinsky and Bock, 2010). Furthermore, unlike the scenario in Ganushchak et al. (2014) study which contained only two entities, the scenarios in our study contained more entities, some of which had contrasting relationships. Therefore, the codability of events in our study was lower, making it more likely for speakers to adopt a linearly incremental framework (Kuchinsky and Bock, 2010). The speech latencies in our study (initial accentuation, 2876 ms; medial accentuation, 3083 ms) were considerably longer than those reported in Ganushchak et al. (2014) (in Chinese: subject focus, 1610 ms; object focus, 1139 ms), indicating greater difficulty in producing focus-accented sentences in our study. The participants in the two studies employed different framework for encoding focus, reflecting the complexity and flexibility of the sentence production process (e.g., Jaeger, 2010; Konopka and Meyer, 2014; Konopka and Kuchinsky, 2015).

In conclusion, this study is the first investigation into the process of producing focus accentuation sentences, shedding light on the processing framework—whether linear incrementality or hierarchical incrementality—is adopted for focus and accentuation processing in speech production. Both behavioral and eye-tracking results consistently supported linear incrementality, indicating that speakers encode information concept-by-concept and word-by-word before articulation, rather than forming a complete conceptual framework and a corresponding sentence-level accentuation pattern. This implies that both conceptualization and prosodic encoding adhere to linear incrementality, thus bridging a gap in the theory of prosodic encoding in sentence production. Moreover, this study is the first instance of participants spontaneously producing sentences with accentuation without information preview, providing a novel approach for future research on focus and accentuation processing in spoken sentence production. Notably, unlike stress at word level, accentuation in different positions can occur simultaneously within the same sentence. Therefore, future research could examine how accentuation in different positions is encoded within the same sentence, providing further insights into the dynamics of sentence production.

## Data Availability

The datasets presented in this study can be found in online repositories. The names of the repository/repositories and accession number(s) can be found at: https://osf.io/nmrqb/?view_only=7e0cc4f2b56044ae9681c21c00dd5bc6.

## References

[B1] Allum P. Wheeldon L. R. (2009). Scope of lexical access in spoken sentence production: implications for the conceptual–syntactic interface. J. Experi. Psychol. Learn. Memory Cogn. 35, 1240–1255. 10.1037/a0016367 19686018

[B2] Aoshima S. Phillips C. Weinberg A. (2004). Processing filler-gap dependencies in a head-final language. J. Mem. Lang. 51, 23–54. 10.1016/j.jml.2004.03.001

[B3] Baayen R. H. Davidson D. J. Bates D. M. (2008). Mixed-effects modeling with crossed random effects for subjects and items. J. Mem. Lang. 59, 390–412. 10.1016/j.jml.2007.12.005

[B4] Barr D. J. Levy R. Scheepers C. Tily H. J. (2013). Random effects structure for confirmatory hypothesis testing: keep it maximal. J. Mem. Lang. 68, 255–278. 10.1016/j.jml.2012.11.001 24403724 PMC3881361

[B5] Bock J. K. Irwin D. E. Davidson D. J. (2004). “Putting first things first,” in: *The Integration of Language, Vision, and Action: Eye Movements and the Visual World*, eds J. Henderson and F. Ferreira (New York, NY: Psychology Press), 249–278.

[B6] Brown-Schmidt S. Konopka A. E. (2008). Little houses and casas pequeñas: Message formulation and syntactic form in unscripted speech with speakers of English and Spanish. Cognition 109, 274–280. 10.1016/j.cognition.2008.07.011 18842259 PMC2665878

[B7] Burght C. Friederici A. D. Goucha T. Hartwigsen G. (2021). Pitch accents create dissociable syntactic and semantic expectations during sentence processing. Cognition 212:104702. 10.1016/j.cognition.2021.104702 33857845

[B8] Chen Y. Xu Y. Guion-Anderson S. (2015). Prosodic realization of focus in bilingual production of Southern Min and Mandarin. Phonetica 71, 249–270. 10.1159/000371891 25997840

[B9] Chomsky N. (1971). “Deep structure, surface structure and semantic interpretation,” in: *Semantics: An Interdisciplinary Reader in Philosophy, Linguistics and Psychology*, eds D. D. Steinberg and L. A. Jakobovits (Cambridge: Cambridge University Press), 183–216.

[B10] Cooper W. E. Danly M. (1981). Segmental and temporal aspects of utterance-final lengthening. Phonetica 38, 106–115. 10.1159/000260017

[B11] Cutler A. (1984). Stress and accent in language production and understanding. Intonat. Accent Rhythm 8, 77–90. 10.1515/9783110863239.77

[B12] Dahan D. Tanenhaus M. K. Chambers C. G. (2002). Accent and reference resolution in spoken-language comprehension. J. Mem. Lang. 47, 292–314. 10.1016/S0749-596X(02)00001-325015025

[B13] Dimitrova D. Stowe L. A. Redeker G. Hoeks J. C. J. (2012). Less is not more: neural responses to missing and superfluous accents in context. J. Cogn. Neurosci. 24, 2400–2418. 10.1162/jocn_a_00302 23016764

[B14] Do M. L. Kaiser E. (2019). Subjecthood and linear order in linguistic encoding: evidence from the real-time production of wh-questions in English and Mandarin Chinese. J. Mem. Lang. 105, 60–75. 10.1016/j.jml.2018.11.001

[B15] Eady S. J. Cooper W. E. (1986). Speech intonation and focus location in matched statements and questions. J. Acoust. Soc. Am. 80, 402–415. 10.1121/1.394091 3745672

[B16] Faul F. Erdfelder E. Buchner A. Lang A. G. (2009). Statistical power analyses using G^*^ Power 3.1: tests for correlation and regression analyses. Behav. Res. Methods 41, 1149–1160. 10.3758/BRM.41.4.1149 19897823

[B17] Ferreira F. (1993). Creation of prosody during sentence production. Psychol. Rev. 100:233. 10.1037/0033-295X.100.2.233 8483983

[B18] Ferreira V. S. (2010). Language production. Wiley Interdisc. Rev. 1, 834–844. 10.1002/wcs.70 26271781

[B19] Ganushchak L. Y. Konopka A. E. Chen Y. (2014). What the eyes say about planning of focused referents during sentence formulation: a cross-linguistic investigation. Front. Psychol. 5:1124. 10.3389/fpsyg.2014.01124 25324820 PMC4183096

[B20] Ganushchak L. Y. Konopka A. E. Chen Y. (2017). Accessibility of referent information influences sentence planning: an eye-tracking study. Front. Psychol. 8:250. 10.3389/fpsyg.2017.00250 28293201 PMC5328952

[B21] Garrett M. (1980). “Levels of processing in sentence production,” in Language Production Vol. 1: Speech and Talk (London: Academic Press), 177–220.

[B22] Garrett M. F. (1975). “The analysis of sentence production,” in Psychology of Learning and Motivation (New York, NY: Academic Press), 133–177. 10.1016/S0079-7421(08)60270-4

[B23] Garrett M. F. (1976). Syntactic processes in sentence production. New Approach. Lang. Mech. 30, 231–256.

[B24] Gee J. P. Grosjean F. (1983). Performance structures: a psycholinguistic and linguistic appraisal. Cogn. Psychol. 15, 411–458. 10.1016/0010-0285(83)90014-2

[B25] Genovese C. R. Lazar N. A. Nichols T. (2002). Thresholding of statistical maps in functional neuroimaging using the false discovery rate. Neuroimage 15, 870–878. 10.1006/nimg.2001.1037 11906227

[B26] Gleitman L. R. January D. Nappa R. Trueswell J. C. (2007). On the give and take between event apprehension and utterance formulation. J. Mem. Lang. 57, 544–569. 10.1016/j.jml.2007.01.007 18978929 PMC2151743

[B27] Griffin Z. (2001). Gaze durations during speech reflect word selection and phonological encoding. Cognition 82, B1–14. 10.1016/S0010-0277(01)00138-X 11672707 PMC5130081

[B28] Griffin Z. M. Bock K. (2000). What the eyes say about speaking. Psychol. Sci. 11, 274–279. 10.1111/1467-9280.00255 11273384 PMC5536117

[B29] Gundel J. K. Hedberg N. Zacharski R. (1993). Cognitive status and the form of referring expressions in discourse. Language 69, 274–307. 10.2307/41653534489770

[B30] Gussenhoven C. (1983). Focus, mode and the nucleus. J. Linguist. 19, 377–417. 10.1017/S0022226700007799

[B31] Hwang H. Kaiser E. (2014). The role of the verb in grammatical function assignment in English and Korean. J. Experi. Psychol. 40:1363. 10.1037/a0036797 24884649

[B32] Ito K. Garnsey S. M. (2004). “Brain responses to focus-related prosodic mismatch in Japanese,” in Proceedings of Speech Prosody, 609–612. 10.21437/SpeechProsody.2004-140

[B33] Jaeger T. F. (2010). Redundancy and reduction: speakers manage syntactic information density. Cogn. Psychol. 61, 23–62. 10.1016/j.cogpsych.2010.02.002 20434141 PMC2896231

[B34] Jeffreys H. (1961). Theory of Probability (3rd ed.). Oxford: Oxford University Press.

[B35] Jescheniak J. D. Schriefers H. Hantsch A. (2003). Utterance format effects phonological priming in the picture-word task: Implications for models of phonological encoding in speech production. J. Experi. Psychol. 29, 441–454. 10.1037/0096-1523.29.2.441 12760627

[B36] Jun S. A. Bishop J. (2015). Priming implicit prosody: prosodic boundaries and individual differences. Lang. Speech 58, 459–473. 10.1177/0023830914563368 27483740

[B37] Jungers M. K. Hupp J. M. (2009). Speech priming: evidence for rate persistence in unscripted speech. Lang. Cogn. Process. 24, 611–624. 10.1080/01690960802602241

[B38] Jungers M. K. Hupp J. M. Dickerson S. D. (2016). Language priming by music and speech: evidence of a shared processing mechanism. Music Percep. 34, 33–39. 10.1525/mp.2016.34.1.33

[B39] Jungers M. K. Palmer C. Speer S. R. (2002). Time after time: the coordinating influence of tempo in music and speech. Cogn. Process. 1, 21–35. Available at: https://citeseerx.ist.psu.edu/document?repid=rep1&type=pdf&doi=6ca6aed947541ccdf73ae079ff3bb86bf4790151

[B40] Klatt H. (1975). Vowel lengthening is syntactically determined in a connected discourse. J. Phon. 3,129–140. 10.1016/S0095-4470(19)31360-9

[B41] Konopka A. E. (2012). Planning ahead: How recent experience with structures and words changes the scope of linguistic planning. J. Mem. Lang. 66, 143–162. 10.1016/j.jml.2011.08.003

[B42] Konopka A. E. Kuchinsky S. E. (2015). How message similarity shapes the time course of sentence formulation. J. Mem. Lang. 84, 1–23. 10.1016/j.jml.2015.04.003

[B43] Konopka A. E. Meyer A. Forest T. A. (2018). Planning to speak in L1 and L2. Cogn. Psychol. 102, 72–104. 10.1016/j.cogpsych.2017.12.003 29407637

[B44] Konopka A. E. Meyer A. S. (2014). Priming sentence planning. Cogn. Psychol. 73, 1–40. 10.1016/j.cogpsych.2014.04.001 24838190

[B45] Kuchinsky S. E. Bock K. (2010). From Seeing to Saying: Perceiving, Planning, Producing. Paper Presented at the 23rd Meeting of the CUNY Human Sentence Processing Conference. New York, NY.

[B46] Kuznetsova A. Brockhoff P. B. Christensen R. H. B. (2017). lmerTest package: tests in linear mixed effects models. J. Stat. Softw. 82:13. 10.18637/jss.v082.i13

[B47] Ladd R. (2008). Intonational Phonology. Cambridge: Cambridge Uniersity Press. 10.1017/CBO9780511808814

[B48] Lee E. Brown-Schmidt S. Watson D. G. (2013). Ways of looking ahead: Hierarchical planning in language production. Cognition 129, 544–562. 10.1016/j.cognition.2013.08.007 24045002 PMC3909534

[B49] Levelt W. J. M. (1989). Speaking: From Intention to Articulation. Cambridge, MA: Bradford/MIT Press. 10.7551/mitpress/6393.001.0001

[B50] Li W. Deng N. Yang Y. Wang L. (2018). Process focus and accentuation at different positions in dialogues: an ERP study. Lang. Cogn. Neurosci. 33, 255–274. 10.1080/23273798.2017.1387278

[B51] Li X. Yang Y. (2013). How long-term memory and accentuation interact during spoken language comprehension. Neuropsychologia 51, 967–978. 10.1016/j.neuropsychologia.2012.12.016 23376769

[B52] Li X. Yang Y. Hagoort P. (2008). Pitch accent and lexical tone processing in Chinese discourse comprehension: an ERP study. Brain Res. 1222, 192–200. 10.1016/j.brainres.2008.05.031 18585687

[B53] Liu F. Xu Y. (2005). Parallel encoding of focus and interrogative meaning in Mandarin intonation. Phonetica 62, 70–87. 10.1159/000090090 16391495

[B54] Magne C. Astésano C. Lacheret-Dujour A. Morel M. Alter K. Besson M. (2005). On-line processing of “pop-out” words in spoken French dialogues. J. Cogn. Neurosci. 17, 740–756. 10.1162/0898929053747667 15904541

[B55] Meyer A. S. (1996). Lexical access in phrase and sentence production: results from picture–word interference experiments. J. Mem. Lang. 35, 477–496. 10.1006/jmla.1996.0026

[B56] Meyer A. S. Meulen F. (2000). Phonological priming effects on speech onset latencies and viewing times in object naming. Psychon. Bull. Rev. 7, 314–319. 10.3758/BF03212987 10909139

[B57] Miyamoto T. Johnson C. (2002). “Accentual phrasing in Japanese: The significance of underlying accents,” in Proceedings of the Speech Prosody (Aix-en-Provence: Laboratoire Parole et Langage), 519–522. 10.21437/SpeechProsody.2002-115

[B58] Momma S. Slevc L. R. Phillips C. (2016). The timing of verb selection in Japanese sentence production. J. Experi. Psychol. Learn. Memory Cogn. 42, 813–824. 10.1037/xlm0000195 26569434

[B59] Morey R. D. Rouder J. N. Jamil T. Urbanek S. Forner K. Ly A. (2022). BayesFactor: Computation of Bayes factors for common designs (Version 0.9.12-4.4). Available at: http://cran.at.r-project.org/web/packages/BayesFactor/index.html (accessed June 2023).

[B60] Patil U. Kentner G. Gollrad A. Kügler F. Féry C. Vasishth S. (2008). Focus, word order and intonation in Hindi. J. South Asian Linguist. 1, 55–72.

[B61] Pierrehumbert J. (1993). Prosody, intonation, and speech technology. Chall. Nat. Lang. Process. 12:11. 10.1017/CBO9780511659478.011

[B62] Schiller N. O. (2006). Lexical stress encoding in single word production estimated by event-related brain potentials. Brain Res. 1112, 201–212. 10.1016/j.brainres.2006.07.027 16893534

[B63] Schiller N. O. Fikkert P. Levelt C. C. (2004). Stress priming in picture naming: an SOA study. Brain Lang. 90, 231–240. 10.1016/S0093-934X(03)00436-X 15172541

[B64] Schiller N. O. Jansma B. M. Peters J. Levelt W. J. (2006). Monitoring metrical stress in polysyllabic words. Lang. Cogn. Process. 21, 112–140. 10.1080/01690960400001861

[B65] Schlenter J. Esaulova Y. Dolscheid S. Penke M. (2022). Ambiguity in case marking does not affect the description of transitive events in German: evidence from sentence production and eye-tracking. Lang. Cogn. Neurosci. 37, 844–865. 10.1080/23273798.2022.2026419

[B66] Schriefers H. (1999). Phonological facilitation in the production of two-word utterances. Eur. J. Cogn. Psychol. 11, 17–50. 10.1080/713752301

[B67] Segaert K. Wheeldon L. Hagoort P. (2016). Unifying structural priming effects on syntactic choices and timing of sentence generation. J. Memory Lang. 2016, 59–80. 10.1016/j.jml.2016.03.011

[B68] Selkirk E. (1995). Sentence prosody: Intonation, stress, and phrasing. Handbook Phonol. Theory 1, 550–569. 10.1111/b.9780631201267.1996.00018.x

[B69] Selkirk E. O. (1984). Phonology and Syntax: The Relation Between Sound and Structure. Cambridge, MA: MIT Press.

[B70] Smith M. Wheeldon L. (1999). High level processing scope in spoken sentence production. Cognition 73, 205–246. 10.1016/S0010-0277(99)00053-0 10585515

[B71] Sussman R. S. Sedivy J. C. (2003). The time-course of processing syntactic dependencies: Evidence from eye-movements. Lang. Cogn. Process. 18, 143–163. 10.1080/01690960143000498

[B72] Tooley K. M. Konopka A. E. Watson D. G. (2014). Can intonational phrase structure be primed (like syntactic structure)? *J. Experi. Psychol*. 40:348. 10.1037/a0034900 24188467 PMC3943528

[B73] Tooley K. M. Konopka A. E. Watson D. G. (2018). Assessing priming for prosodic representations: speaking rate, intonational phrase boundaries, and pitch accenting. Mem. Cognit. 46, 625–641. 10.3758/s13421-018-0789-5 29349696 PMC5943085

[B74] van de Velde M. Meyer A. S. Konopka A. E. (2014). Message formulation and structural assembly: describing “easy” and “hard” events with preferred and dispreferred syntactic structures. J. Mem. Lang. 71, 124–144. 10.1016/j.jml.2013.11.001

[B75] Wheeldon L. Lahiri A. (1997). Prosodic units in speech production. J. Mem. Lang. 37, 356–381. 10.1006/jmla.1997.2517

[B76] Winkler S. (2019). “Ellipsis and prosody,” in: *The Oxford Handbook of Ellipsis* (eds) J. van Craenenbroeck and T. Temmerman (Oxford: Oxford University Press), 356–386. 10.1093/oxfordhb/9780198712398.013.15

[B77] Xu Y. (1999). Effects of tone and focus on the formation and alignment of f0 contours. J. Phon. 27, 55–105. 10.1006/jpho.1999.0086

[B78] Xu Y. Chen S. W. Wang B. (2012). Prosodic focus with and without post-focus compression: A typological divide within the same language family? Linguistic Rev. 29, 131–147. 10.1515/tlr-2012-0006

[B79] Yu W. Chien Y. Wang B. Zhao J. Li W. (2024). The effects of word and beat priming on Mandarin lexical stress recognition: an event-related potential study. Lang. Cogn. 2024, 1–23. 10.1017/langcog.2023.75

[B80] Zhang N. Zhang Q. (2019). Rhythmic pattern facilitates speech production: an ERP study. Sci. Rep. 9, 1–11. 10.1038/s41598-019-49375-8 31506472 PMC6736834

[B81] Zhang Q. Yang Y. (2003). The determiners of picture-naming latency. Acta Psychol. Sinica 35, 447–454. 10.3724/SP.J.1041.2024.0044737113526

